# Real‐World Impact of Olaparib Exposure in Advanced Pancreatic Cancer Patients Harboring Germline *BRCA*1‐2 Pathogenic Variants

**DOI:** 10.1002/cam4.70364

**Published:** 2025-01-24

**Authors:** Michele Milella, Giulia Orsi, Mariacristina di Marco, Lisa Salvatore, Letizia Procaccio, Silvia Noventa, Silvia Bozzarelli, Ingrid Garajova, Enrico Vasile, Guido Giordano, Marina Macchini, Alessandro Cavaliere, Marina Gaule, Francesca Bergamo, Marta Chiaravalli, Andrea Palloni, Riccardo Carloni, Alessandro Bittoni, Monica Niger, Ilario Giovanni Rapposelli, Maria Grazia Rodriquenz, Mario Scartozzi, Stefania Mosconi, Elisa Giommoni, Ilaria Bernardini, Chiara Paratore, Andrea Spallanzani, Katia Bencardino, Laura Forti, Emiliano Tamburini, Sara Lonardi, Aldo Scarpa, Stefano Cascinu, Giampaolo Tortora, Isabella Sperduti, Michele Reni

**Affiliations:** ^1^ Department of Engineering for Innovation Medicine (DIMI), Specialty School in Medical Oncology and Section of Innovation Biomedicine—Oncology Area Faculty of Medicine and Surgery, University of Verona Verona Italy; ^2^ Division of Oncology Verona University and Hospital Trust (AOUI Verona) Verona Italy; ^3^ Department of Medical Oncology IRCCS Ospedale San Raffaele Milan Italy; ^4^ Department of Medical Oncology Università Vita‐Salute San Raffaele Facoltà di Medicina e Chirurgia Milan Italy; ^5^ Department of Medical and Surgical Sciences Alma Mater Studiorum University of Bologna Bologna Italy; ^6^ Medical Oncology Unit IRCSS Azienda Ospedaliero Universitaria di Bologna Bologna Italy; ^7^ Medical Oncology Università Cattolica del Sacro Cuore Facoltà di Medicina e Chirurgia Rome Italy; ^8^ Medical Oncology Fondazione Policlinico Universitario Agostino Gemelli IRCCS Rome Italy; ^9^ Department of Oncology Veneto Institute of Oncology IRCCS Padova Italy; ^10^ Medical Oncology Fondazione Poliambulanza Istituto Ospedaliero Brescia Italy; ^11^ IRCCS Humanitas Research Hospital Milan Italy; ^12^ Medical Oncology Unit University Hospital of Parma Parma Italy; ^13^ Unit Medical Oncology Azienda Ospedaliero‐Universitaria Pisana, Oncology Pisa Italy; ^14^ Policlinico Riuniti di Foggia Unit of Medical Oncology and Biomolecular Therapy Foggia Italy; ^15^ Department of Medical and Surgical Sciences University of Foggia Foggia Italy; ^16^ Oncology Unit Azienda Ospedaliero‐Universitaria Ospedali Riuniti Umberto I—GM Lancisi—G Salesi di Ancona Ancona Italy; ^17^ Medical Oncology Department Fondazione IRCCS Istituto Nazionale Dei Tumori di Milano Milan Italy; ^18^ Department of Medical Oncology IRCCS Istituto Romagnolo per Lo Studio Dei Tumori “Dino Amadori”—IRST Meldola Italy; ^19^ Oncology Unit Ospedale IRCCS Casa Sollievo Della Sofferenza San Giovanni Rotondo (FG) Italy; ^20^ Medical Oncology University and University Hospital Cagliari Italy; ^21^ Oncology Unit ASST Papa Giovanni XIII Bergamo Italy; ^22^ Medical Oncology Division Azienda Ospedaliero‐Universitaria Careggi Firenze Italy; ^23^ Medical Oncology Unit Ospedale Ramazzini Carpi (MO) Italy; ^24^ Department of Oncology University of Turin, Ordine Mauriziano Hospital Turin Italy; ^25^ Department of Oncology and Hematology University Hospital of Modena Modena Italy; ^26^ Niguarda Cancer Center Grande Ospedale Metropolitano Niguarda Milan Italy; ^27^ Medical Oncology Division Azienda Ospedaliero‐Universitaria Maggiore Della Carità Novara Italy; ^28^ Medical Oncology and Palliative Care Department Azienda Ospedaliera Cardinale G. Panico Lecce Italy; ^29^ ARC‐Net Research Centre and Department of Diagnostics and Public Health—Section of Pathology University of Verona Verona Italy; ^30^ Clinical Trial Center—Biostatistics & Bioinformatics IRCCS—Regina Elena National Cancer Institute Rome Italy

**Keywords:** germline *BRCA*1/2 pathogenic variants, metastatic, olaparib, pancreatic cancer, poly(ADP‐ribose) polymerase inhibitors, real‐world experience, survival analysis

## Abstract

**Introduction:**

Pancreatic cancer arising in the context of *BRCA* predisposition may benefit from poly(ADP‐ribose) polymerase inhibitors. We analyzed real‐world data on the impact of olaparib on survival in metastatic pancreatic cancer patients harboring germline *BRCA* mutations in Italy, where olaparib is not reimbursed for this indication.

**Methods:**

Clinico/pathological data of pancreatic cancer patients with documented *BRCA*1‐2 germline pathogenic variants who had received first‐line chemotherapy for metastatic disease were collected from 23 Italian oncology departments and the impact of olaparib exposure on overall survival (OS) was analyzed.

**Results:**

Of 114, 53 *BRCA*‐mutant pancreatic cancer patients had received olaparib for metastatic disease. OS was significantly longer in patients who were exposed to olaparib (hazard ratio [HR] 0.568, 95% confidence interval [CI] 0.351–0.918, log‐rank *p* = 0.02) in any setting/line of treatment; similar results were obtained for patients who received olaparib as maintenance treatment (in any line of treatment), patients who had stage IV disease at diagnosis, and patients who did not experience progressive disease as their best response to first‐line chemotherapy. Exposure to olaparib in the first‐line maintenance setting after platinum‐based chemotherapy, however, did not significantly impact survival. At multivariate analysis, CA19.9 levels at diagnosis and response to first‐line chemotherapy were independently prognostic; however, when response to chemotherapy was excluded, any exposure to olaparib was a significant independent predictor of longer OS, together with CA19.9 levels.

**Conclusion:**

The real‐world data presented here support the use of olaparib for metastatic disease in germline *BRCA*‐mutant pancreatic cancer patients, as it may significantly prolong survival.

## Abstract 2

In a real‐world population of Italian patients with advanced pancreatic cancer carrying germline *BRCA* pathogenic variants, exposure to olaparib resulted in significantly longer overall survival. However, the prognostic impact of olaparib exposure was not evident in the first‐line maintenance setting, after platinum‐based chemotherapy.

## 1 Introduction

Pancreatic cancer is the seventh leading cause of cancer‐related deaths, accounting for 4.5% of cancer mortality worldwide [1]. In Italy, pancreatic cancer is estimated to have caused 12,900 deaths in 2021 [2] and both incidence and mortality are projected to substantially increase in the next 20 years [3]. Despite progresses, 5‐year survival for pancreatic cancer is the lowest among solid tumors (11% and 12% in men and women in Italy, respectively) with an estimated cure rate < 4% in both sexes [2].

Loss‐of‐function alterations in *BRCA1* and/or *BRCA2* genes impair repair of DNA double‐strand breaks, leading to genomic instability and malignant transformation; germline *BRCA*1‐2 pathogenic variants (g*BRCA*1‐2pv) are indeed associated with increased risk of developing ovarian, breast, prostate, and pancreatic cancers [4, 5, 6]. Poly(ADP‐ribose) polymerase inhibitors (PARPi) prevent the repair of DNA single‐strand breaks, leading to the accumulation of damaged DNA, and their mechanism of action can be exploited to produce synthetic lethality in tumors harboring homologous recombination repair defects (HRD), such as those arising in g*BRCA*1‐2pv carriers [7]. In Italy, g*BRCA*1‐2pv are found in approximately 8% of patients affected by pancreatic ductal adenocarcinoma (PDAC) [8]. In these patients, platinum‐based combination chemotherapy has shown substantial activity [9, 10]. Moreover, the Pancreas cancer OLaparib Ongoing (POLO) trial has demonstrated the efficacy of the PARPi olaparib as maintenance therapy in patients with g*BRCA*1‐2pv and metastatic PDAC, whose disease had not progressed on first‐line platinum‐based chemotherapy [11]. The trial met its primary endpoint of demonstrating a statistically significant and clinically meaningful improvement in progression‐free survival (PFS) with olaparib maintenance: PFS was 7.4 months in the olaparib arm versus 3.8 months in the placebo arm (hazard ratio [HR] 0.53, 95% confidence interval [CI] 0.35–0.82, *p* = 0.004). Although no significant difference in median overall survival (OS) was observed, the estimated 3‐year survival after random assignment was 33.9% versus 17.8% in the olaparib and placebo arms, respectively [12]; moreover, a number of clinically relevant secondary endpoints, such as median time to first subsequent cancer therapy or death (HR 0.44, 95% CI 0.30–0.66, *p* < 0.0001), time to second subsequent cancer therapy or death (HR 0.61, 95% CI 0.42–0.89, *p* = 0.0111), and time to discontinuation of study treatment or death (HR 0.43, 95% CI 0.29–0.63, *p* < 0001), also significantly favored olaparib, resulting in FDA and EMA approval in this indication. Despite regulatory approval and endorsement by major guidelines (NCCN, ASCO, AIOM), olaparib is currently not reimbursed by the Italian National Health System (NHS) for metastatic PDAC based on the lack of statistically significant OS benefit.

Taking advantage of an ongoing Italian multicentric collaborative effort, we set out to collect and analyze real‐world data on olaparib's impact on OS in g*BRCA*1‐2pv patients undergoing treatment for metastatic PDAC.

## 2 Materials and Methods

This retrospective multicenter study involved 23 Italian oncology departments and was based on clinico/pathological data retrieved from medical records and collected in an electronic database. Patients of any age with documented g*BRCA*1‐2pv were eligible for this analysis if they had a pathologically confirmed diagnosis of PDAC, had received first‐line chemotherapy for metastatic disease, irrespective of the regimen, and had available data on exposure to olaparib and long‐term outcome. All patients enrolled in the study provided a written informed consent for germline *BRCA*1‐2 testing, which included the authorization for the use of clinico/pathological and genomic data for scientific purposes, in full compliance with privacy policy.

Patients and tumor characteristics included type of g*BRCA*1‐2pv, age, clinical stage (AJCC/UICC TNM 8th Edition, 2017) at diagnosis, presence/absence of liver metastases, levels of carbohydrate antigen 19.9 (CA19.9) at diagnosis, previous surgery, regimen and setting (adjuvant, neoadjuvant/primary, first, second, third, or subsequent lines for metastatic disease) of the chemotherapy received, response to first‐line chemotherapy, exposure to olaparib (yes/no), setting of exposure to olaparib (first‐line maintenance, second‐line, second‐line maintenance, third or subsequent lines, third‐ or subsequent‐line maintenance), and outcome (OS). OS was calculated from the date of first‐line chemotherapy start until the date of death or last follow‐up visit. Molecular analysis of *BRCA1* and *BRCA2* included sequencing of the whole coding regions and intronic junctions, as well as multiplex ligation‐dependent probe amplification analysis for detection of large intragenic deletions/duplications; classification of variants was carried out in agreement with the American College of Medical Genetics and Genomics and the Association for Molecular Pathology and Enhancing Neuro Imaging Genetics through Meta‐Analysis (ENIGMA) criteria (www.enigmaconsortium.org), as previously reported [8]. Activity, tolerability, and long‐term outcomes of first‐ and second‐line chemotherapy on a largely overlapping cohort of advanced PDAC patients, who were tested for g*BRCA*1‐2pv in Italy, have also been previously reported by our group [10, 13]. In the population analyzed (PDAC patients who were screened for g*BRCA*1‐2pv between 2015 and 2021), olaparib was made available through either participation into clinical trials or atypical drug access schemes (named patient programs, 5% fund of the Italian Drug Agency—AIFA, hospital‐supported off‐label use); currently, olaparib is not reimbursed by the Italian NHS for metastatic PDAC.

### 2.1 Statistical Analysis

Data were summarized using basic descriptive statistics. The Mann–Whitney *U* test was used for comparing quantitative variables. The chi‐square test or Fisher's exact test were used for categorical variables, when appropriate. HRs with 95% CIs were estimated for OS. A *p* < 0.05 was considered significant. Variables found to be significant at univariate analysis were included in the multivariate analysis. A multivariate logistic regression and proportional hazard model were developed using stepwise regression (forward selection, enter limit and remove limit, *p* = 0.10 and *p* = 0.15, respectively), to identify independent predictors of OS. Interactions between significant investigational variables were taken into account when developing the multivariate model. OS curves were generated using the Kaplan–Meier method. Log‐rank test was used to evaluate differences between curves. Statistical analysis was performed by using the SPSS 29.0 package (SPSS Inc., Chicago, IL, USA) for Windows.

## 3 Results

We retrospectively collected clinical data of a cohort of 114 g*BRCA*1‐2pv PDAC patients undergoing treatment for metastatic disease at different Italian institutions as part of a multicentric collaborative effort; clinical characteristics of such patient cohort are reported in Table 1 and a flowchart relative to patients' selection criteria for each individual comparison is shown in Figure S1. Of 114 PDAC patients, 53 (46%) received olaparib as first‐line maintenance in 37 (70%) of patients, second‐line or maintenance treatment in the absence of progressive disease (PD) to second‐line chemotherapy (second‐line maintenance) in 7 (13%), and third or subsequent treatment lines in 9 (17%; only in 1 patient as maintenance treatment in the absence of PD to third‐line chemotherapy) (Table 2). Clinical characteristics of patients who did or did not receive olaparib were well balanced, except for median levels of CA19.9 at diagnosis (significantly lower in patients who received olaparib, *p* = 0.043) and exposure to platinum‐containing chemotherapy (significantly less patients who did not receive olaparib had been exposed to platinum [in any line of treatment or as first line, *p* < 0.0001 for both comparisons; Table 1]). Median follow‐up was significantly longer in patients receiving olaparib in any line of treatment (*p* < 0.0001; Table 1).

**TABLE 1:** Comparison of clinicopathologic characteristics of pancreatic ductal adenocarcinoma (PDAC) patients carrying g*BRCA*1‐2pv who did or did not receive olaparib for metastatic disease.

Patients' characteristics	Entire population *n* (%)	Olaparib		*p*
		Yes (*n* = 53)	No (*n* = 61)	
Age (years)	Median: 59	Median: 57	Median: 59	n.s.
	Range: 34–84	Range: 35–76	Range: 34–84	
Gender				
M	51 (45)	25 (47)	26 (43)	n.s.
F	63 (55)	28 (53)	35 (57)	
Stage at diagnosis				
I	5 (4)	3 (6)	2 (3)	n.s.
II	13 (11)	7 (13)	6 (10)	
III	9 (8)	3 (6)	6 (10)	
IV	87 (76)	40 (75)	47 (77)	
Liver metastases				
Yes	43 (38)	21 (40)	22 (36)	n.s.
No	69 (60)	31 (58)	38 (62)	
N/A	2 (2)	1 (2)	1 (2)	
g*BRCA* mutations				
*BRCA*1	27 (24)	11 (21)	16 (26)	n.s.
*BRCA*2	85 (75)	40 (75)	45 (74)	
*BRCA*1+2	2 (2)	2 (4)	0 (0)	
Previous surgery				
Yes	33 (29)	17 (32)	16 (26)	n.s.
No	81 (71)	36 (68)	45 (74)	
Neoadjuvant chemotherapy				
Yes	16 (14)	6 (11)	10 (16)	n.s.
No	98 (86)	47 (89)	51 (84)	
Adjuvant chemotherapy				
Yes	13 (11)	8 (15)	5 (8)	n.s.
No	101 (89)	45 (85)	56 (92)	
Platinum‐based chemotherapy				
Any^a^	100 (88)	53 (100)	47 (77)	< 0.0001
First line	75 (66)	45 (85)	30 (49)	< 0.0001
Second or subsequent	20 (17)	7 (13)	13 (21)	0.326
No platinum	14 (12)	0 (0)	14 (23)	0.002
CA19.9 levels at diagnosis (IU/L)	Median: 475	Median: 238	Median: 815	0.043
	Range: 0.7–456,308	Range: 0.7–104,429	Range: 0.8–456,308	
Olaparib exposure				
Any	53 (46)	53 (46)	n.a.	n.a.
First‐line maintenance	37 (32)	37 (32)		
Any maintenance	43 (38)	43 (38)		
Follow‐up (months)^b^	Median: 16.5	Median: 21.6	Median: 11.2	< 0.0001
	Range: 2–156	Range: 2–156	Range: 2–106	

^a^ Five patients received platinum‐based chemotherapy as either adjuvant (*n* = 2) or neoadjuvant (*n* = 5, two of whom also received platinum‐based adjuvant) treatment, but not for metastatic disease (one in the group that went on to receive olaparib, four in the group that did not receive olaparib).

^b^ Follow‐up was calculated from the start of first‐line chemotherapy.

Abbreviations: n.a., not applicable; n.s., not significant.

**TABLE 2:** Treatment phase in which olaparib was administered.

	*n* (%)^a^
First‐line maintenance	37 (70)
Second‐line	2 (4)
Second‐line maintenance	5 (9)
Third‐ or subsequent	8 (15)
Third‐line maintenance	1 (2)
Total number of patients receiving olaparib (any treatment line)	53/114 (46%)

^a^ Percentages are calculated out of the total number of patients receiving olaparib (*n* = 53), unless otherwise indicated.

**FIGURE 1:**
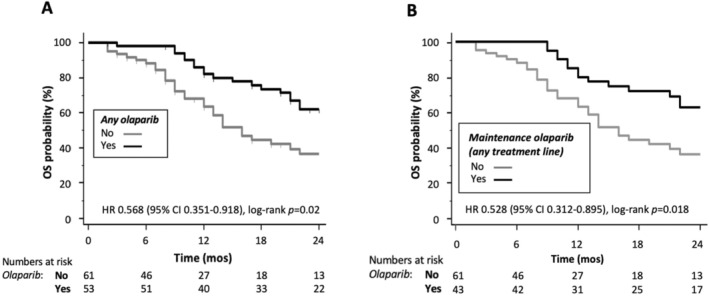
Impact of olaparib exposure on overall survival (OS). (A) Kaplan–Meier curves for OS for the entire population of g*BRCA*1‐2pv pancreatic ductal adenocarcinoma (PDAC) patients (*n* = 114), according to having (*yes*, black line) or not having (*no*, gray line) received olaparib, are shown; hazard ratio (HR) with 95% confidence interval (CI) and statistical significance of the differences between curves according to log‐rank test are reported. (B) Kaplan–Meier curves comparing g*BRCA*1‐2pv PDAC patients who received maintenance olaparib in any line of treatment in the absence of progression to the previous line of chemotherapy (*yes*, black line; *n* = 43) or did not receive any olaparib (*no*, gray line; *n* = 61) are shown; HR with 95% CI and statistical significance of the differences between curves according to log‐rank test are reported.

In the entire cohort of patients (*n* = 114), OS was significantly longer in patients who were exposed to olaparib (HR 0.568, 95% CI 0.351–0.918, log‐rank *p* = 0.02; Figure 1A and Table S1); a similar impact on OS was evident when considering patients who received olaparib as maintenance treatment in the absence of PD to chemotherapy in any line of treatment (Figure 1B).

**FIGURE 2:**
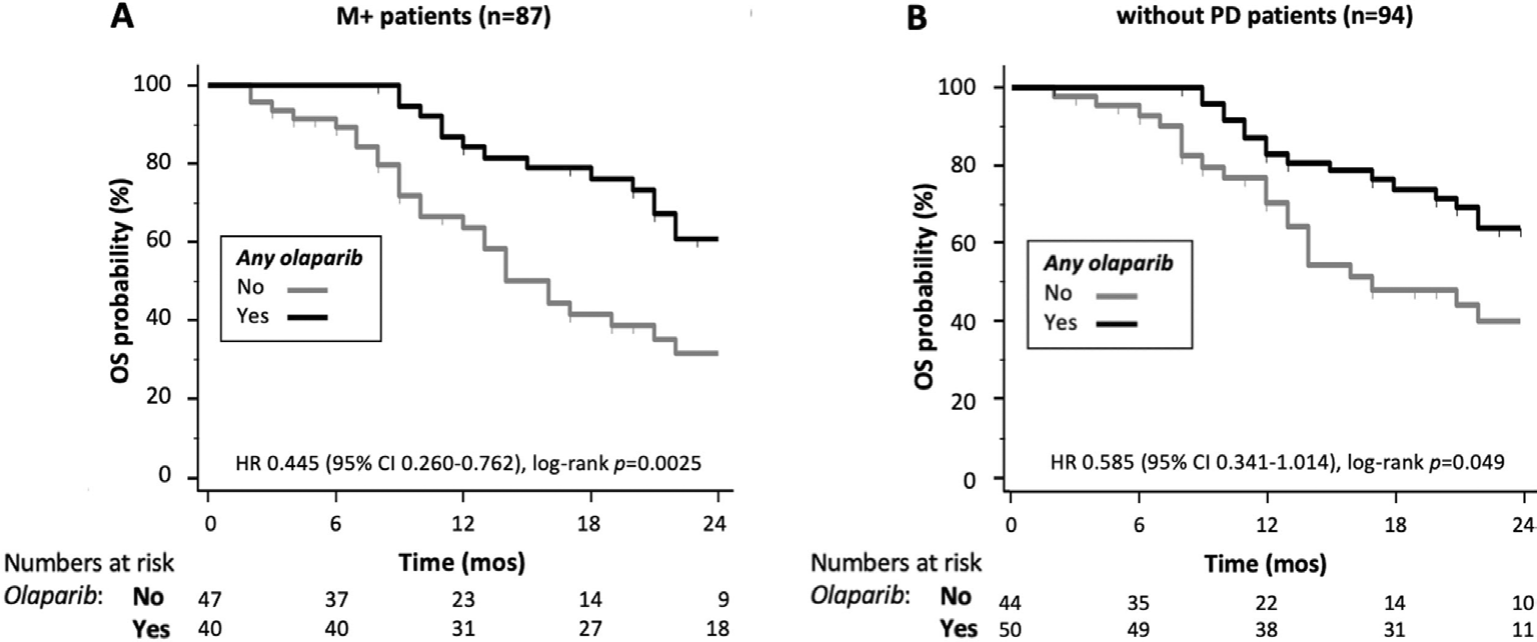
Impact of olaparib exposure on overall survival (OS) in subgroups of patients with metastatic disease at diagnosis (M+) or without progressive disease (PD) as best response to first‐line chemotherapy. (A) Kaplan–Meier curves for OS relative to g*BRCA*1‐2pv pancreatic ductal adenocarcinoma (PDAC) patients with stage IV disease at the time of diagnosis (M+, *n* = 87), according to having (*yes*, black line) or not having (*no*, gray line) received olaparib, are shown; hazard ratio (HR) with 95% confidence interval (CI) and statistical significance of the differences between curves according to log‐rank test are reported. (B) Kaplan–Meier curves for OS relative to g*BRCA*1‐2pv PDAC patients who did not experience PD as their best response to first‐line chemotherapy (*n* = 73; of whom, 51 received platinum‐based first‐line chemotherapy), according to having (*yes*, black line) or not having (*no*, gray line) received olaparib, are shown; HR with 95% CI and statistical significance of the differences between curves according to log‐rank test are reported.

Significant predictors of longer OS at univariate analysis in the entire population are shown in Table S2 and included CA19.9 levels below the median, previous surgery, exposure to olaparib (either any exposure or exposure in the maintenance setting), and response (complete response [CR]/partial response [PR] vs. stable disease [SD] or PD) to first‐line chemotherapy; of these, CA19.9 levels, surgery, and response to first‐line chemotherapy maintained their independent prognostic value at multivariate analysis (Table S2). Given the strong independent prognostic power of previous surgery, we concentrated our prognostic analysis on the 87 patients who were diagnosed with metastatic disease and therefore did not undergo surgery (M+; Table 3); CA19.9 levels below the median, exposure to olaparib (any treatment line, *p* = 0.003, and any maintenance, *p* = 0.009), and response (CR/PR vs. SD or PD) to first‐line chemotherapy were significant predictors of longer OS at univariate analysis. When all significant factors were included in the multivariate model (model 1 in Table 3), only CA19.9 and response to chemotherapy retained their independent prognostic value; however, when response to first‐line chemotherapy was excluded from multivariate analysis, any exposure to olaparib (but not exposure to maintenance olaparib) was a significant independent predictor of longer OS, together with CA19.9 levels (model 2 in Table 3). Consistently, exposure to olaparib significantly impacted on the survival of patients who were diagnosed with stage IV disease (M+, *n* = 87; Figure 2A and Table S1) and of patients who did not experience PD as their best response to first‐line chemotherapy (without PD, *n* = 94; Figure 2B and Table S1). Conversely, differences in survival favoring M+/without PD patients exposed to olaparib were only of borderline significance (*n* = 73, of whom 51 received platinum‐based first‐line chemotherapy; Figure S2A and Table S1) and did not significantly differ in patients who received olaparib in its current indication (first‐line maintenance in patients not progressing after platinum‐based chemotherapy, *n* = 60; Figure S2B). Finally, 10 patients received olaparib as second (*n* = 2, Table 2) or third and further (*n* = 8) line of systemic treatment for metastatic disease, immediately after progressing to the previous chemotherapy line; characteristics of this small subgroup of patients are described in Table S3 and their median OS (34 months, 95% CI: 3–48 months) was similar to that of the entire population receiving olaparib (Figure S3).

**TABLE 3:** Univariate and multivariate Cox regression analysis for overall survival (OS) in patients with metastatic disease at diagnosis.

Factor	Univariate		Multivariate *model* 1 (*including response to first‐line CHT*)		Multivariate *model 2* (*not including response to first‐line CHT*)	
	HR (95% CI)	*p*	HR (95% CI)	*p*	HR (95% CI)	*p*
Age (≤ 59 vs. > 59 years)	0.748 (0.437–1.280)	0.289	—		—	
Gender (female vs. male)	0.988 (0.579–1.687)	0.965	—		—	
*CA19.9* (*below median* vs. *above*)	*0.422* (*0.231*–*0.773*)	*0.005*	*0.536* (*0.288*–*0.998*)	*0.049*	*0.454* (*0.245*–*0.841*)	*0.012*
Platinum first line (yes vs. no)	0.711 (0.417–1.211)	0.209	—		—	
Platinum any line (yes vs. no)	0.722 (0.340–1.535)	0.397	—		—	
*Any olaparib* (*yes* vs. *no*)	*0.445* (*0.260*–*0.762*)	*0.003*	—		*0.527* (*0.302*–*0.921*)	*0.024*
*Maintenance olaparib* (*yes* vs. *no*)^a^	*0.467* (*0.263*–*0.829*)	*0.009*	—		—	
*Response to first‐line*	—	*< 0.0001*	—	*0.001*	*n.a*.	*n.a*.
*CR/PR* versus *PD*	*0.393* (*0.208*–*0.742*)	*0.004*	*0.240* (*0.114*–*0.509*)	*< 0.0001*		
*SD* versus *PD*	*0.708* (*0.324*–*1.550*)	*0.388*	*0.493* (*0.219*–*1.110*)	*0.088*		
*CR/PR* versus *SD*	*0.278* (*0.136*–*0.568*)	*< 0.0001*	*0.488* (*0.250*–*0.952*)	*0.035*		

Abbreviations: 95% CI, 95% confidence interval; CHT, chemotherapy; CR, complete response; HR, hazard ratio; *n.a*., not applicable; PD, progressive disease; PR, partial response; SD, stable disease. *Note:* Italics was used to highlight significant variables.

^a^ Patients receiving olaparib as maintenance therapy in the absence of progression to chemotherapy in any treatment line (*n* = 28) were considered.

Exploratory analysis of the prognostic/predictive impact of g*BRCA1*pv versus g*BRCA2*pv revealed no significant difference in the overall population (*p* = 0.35 and *p* = 0.14 by log‐rank and Tarone–Ware tests, respectively), regardless of whether patients had (*p* = 0.63 and *p* = 0.36 by log‐rank and Tarone–Ware tests, respectively) or had not received olaparib (*p* = 0.44 and *p* = 0.34 by log‐rank and Tarone–Ware tests, respectively; data not shown).

## 4 Discussion

In this retrospective analysis of real‐world data on metastatic PDAC patients carrying g*BRCA*1‐2pv, exposure to olaparib, regardless of the treatment line or setting, was associated with an increase in OS, which was both statistically significant and clinically meaningful. To our knowledge this is the first real‐world evidence of a potential survival benefit of a PARPi in advanced PDAC patients carrying g*BRCA*1‐2pv, obtained in the Italian setting, in which olaparib is only available through atypical drug access schemes (named patient programs, off‐label use, etc.). The POLO trial failed to demonstrate a statistically significant OS advantage for metastatic g*BRCA*1‐2pv PDAC patients not progressing after at least 16 weeks of a platinum‐based first‐line regimen and receiving maintenance olaparib, as compared to placebo [12]. Potential reasons behind the observed lack of OS advantage are subject of intense biological and clinical speculation, including subsequent exposure to PARPi of patients initially randomized in the placebo arm: overall, a total of 109 patients (92 initially randomized to olaparib and 17 [27%] of 62 initially randomized to placebo who received a PARPi in further treatment lines, after progression on placebo), accounting for 71% of the entire trial population, were exposed to a PARPi during the course of metastatic disease, making it difficult to assess olaparib's impact on OS [12].

Selection biases, intrinsic to the retrospective nature of a real‐world data collection, may explain discrepancies in olaparib's impact on the OS of g*BRCA*1‐2pv PDAC patients analyzed in our experience, as compared to the POLO trial population. Indeed, in our experience, median CA19.9 levels were lower in patients who had received olaparib and significantly more patients in the olaparib group had previously been exposed to platinum (either in first or in any treatment line). Albeit coming from retrospective/real‐world experiences, the potential impact of platinum exposure on the OS of advanced PDAC patients carrying g*BRCA*1‐2pv [9] or whose tumors display HRD (particularly germline or somatic *BRCA1/2* and *PALB2* mutations) [14] is well known and has recently been confirmed by our own data in g*BRCA*1‐2pv both in first and second line, favoring the use of multiagent regimens (platinum‐containing triplets and quadruplets, as opposed to doublets) and as early as possible in the course of metastatic disease (first, as opposed to second or subsequent, line[s]) [10, 13]. Response to platinum is also of great importance in predicting sensitivity to PARPi [15, 16, 17]; given the overlap in the biologic mechanisms of susceptibility and resistance to platinum and PARPi, resistance to platinum‐based chemotherapy is generally considered predictive of resistance to PARPi treatment [18, 19, 20], though a clinically solid definition of platinum sensitivity/resistance in PDAC is currently lacking. This might explain why, in our analysis, the OS impact of platinum response (particularly the occurrence of CR/PR) obscures that of olaparib exposure in multivariate analysis; if response to platinum is not considered in the prognostic model, exposure to olaparib (in any treatment setting) remains a significant predictor of longer OS at multivariate analysis. Consistent with the results reported by the POLO trial in pancreatic cancer [11] and those reported in other *BRCA*‐related malignancies [21, 22, 23], no significant differences in OS were observed in our series when comparing g*BRCA1*pv and g*BRCA2*pv, regardless of whether patients had or had not received olaparib (data not shown). Besides the current approach of using response to platinum as a mean to select patients who may benefit most from PARPi, the issue of identifying reliable, biology‐based, pretreatment biomarkers of response to platinum and/or PARPi remains open; whether encompassing parallel germline and somatic sequencing to identify biallelic gene inactivation, signature‐based definition(s) of HRD, or functional testing (e.g., by qualitative and quantitative RAD51 foci formation) [24, 25, 26], this represents the top translational research priority in the field.

At a closer look, when we progressively restricted the analysis to a population that had received olaparib in a setting closer to that of the POLO trial (M+/no PD, first‐line maintenance; Figure S2), the impact of olaparib on OS was progressively lost; along these lines, having received olaparib as maintenance in the absence of PD to chemotherapy (in any treatment line) was not an independent predictor of longer OS, even when response to chemotherapy was not considered (multivariate model 2 in Table 3); however, overall our data support the importance of exposing g*BRCA*1‐2pv advanced PDAC patients to olaparib, at any point during their metastatic disease course, as it may confer clinical and possibly OS benefit. This raises the interesting biological and clinical question of whether the best sequencing option for PARPi in PDAC is their use directly after a platinum‐based regimen, as first‐line maintenance. Even though this approach is largely supported by clinical data in ovarian cancer, in breast and prostate cancer (the other approved indications of PARPi) exposure and/or response to platinum is not a prerequisite for PARPi use [27, 28]. Since molecular mechanisms of acquired resistance to platinum compounds and PARPi are partially overlapping [17, 29], one might speculate that while chemonaïve, platinum‐sensitive, g*BRCA*‐defective cancer cells are characterized by somatic loss of heterozygosity (LOH) of the remaining allele, platinum exposure could select out preexisting BRCA‐proficient cells (so‐called “restoration of heterozygosity”) [30], which in turn would be cross‐resistant to both platinum and PARPi and cause relatively rapid disease progression upon maintenance treatment; conversely, a platinum‐free interval may potentially allow cells carrying *BRCA* LOH to outcompete BRCA‐proficient cells, thereby restoring functional BRCA‐deficiency and potential sensitivity to platinum compounds and PARPi. Thus, from a clinical standpoint, not using a PARPi immediately after platinum might theoretically be advantageous. A variable proportion of BRCA‐mutant PDAC patients is treated with PARPi after progression to a platinum‐containing regimen in real world (e.g., 28% in ref. [31] and 19% in our experience) and unexpected responses/prolonged disease control are occasionally reported [32, 33, 34, 35, 36]; in our series, median OS from the start of first‐line systemic treatment for metastatic disease of the 10 patients who received olaparib straight after progressing on the previous chemotherapy line was not different from that of the entire population of patients receiving olaparib (34 months). Interestingly, most of these unexpected responses occur in patients who are treated with PARPi not immediately after progressing on a platinum‐containing regimen, but after one or two intervening, nonplatinum‐containing, treatment lines [32, 33, 34, 35, 36]. Albeit intriguing mechanistically, this hypothesis remains speculative at present and the most likely explanation for the lack of OS benefit in the POLO trial remains a potential confounding effect of crossover to PARPi in subsequent treatment lines in the placebo group (see before).

## 5 Conclusions

Despite limitations, the real‐world data presented here strongly support the importance of exposing g*BRCA*1‐2pv advanced PDAC patients to olaparib at any point during their metastatic disease course, as it confers substantial clinical and possible OS benefit. Although alternative treatment sequencing may speculatively be explored, first‐line maintenance should be pursued as the setting currently most supported by scientific evidence [12, 37].

## Author Contributions

**Michele Milella:** conceptualization (lead), data curation (equal), formal analysis (lead), funding acquisition (lead), investigation (equal), methodology (lead), supervision (lead), writing – original draft (lead), writing – review and editing (lead). **Giulia Orsi:** conceptualization (equal), data curation (lead), formal analysis (lead), investigation (equal), methodology (equal), writing – original draft (lead), writing – review and editing (lead). **Mariacristina di Marco:** conceptualization (equal), data curation (equal), formal analysis (equal), investigation (equal), writing – review and editing (equal). **Lisa Salvatore:** conceptualization (equal), data curation (equal), formal analysis (equal), investigation (equal), writing – review and editing (equal). **Letizia Procaccio:** conceptualization (equal), data curation (equal), formal analysis (equal), investigation (equal), writing – review and editing (equal). **Silvia Noventa:** conceptualization (equal), data curation (equal), formal analysis (equal), investigation (equal), writing – review and editing (equal). **Silvia Bozzarelli:** conceptualization (equal), data curation (equal), formal analysis (equal), investigation (equal), writing – review and editing (equal). **Ingrid Garajova:** conceptualization (equal), data curation (equal), formal analysis (equal), investigation (equal), writing – review and editing (equal). **Enrico Vasile:** conceptualization (equal), data curation (equal), formal analysis (equal), investigation (equal), writing – review and editing (equal). **Guido Giordano:** conceptualization (equal), data curation (equal), formal analysis (equal), investigation (equal), writing – review and editing (equal). **Marina Macchini:** conceptualization (equal), data curation (equal), formal analysis (equal), writing – review and editing (equal). **Alessandro Cavaliere:** conceptualization (equal), data curation (equal), formal analysis (equal), investigation (equal), writing – review and editing (equal). **Marina Gaule:** conceptualization (equal), data curation (equal), formal analysis (equal), investigation (equal), writing – review and editing (equal). **Francesca Bergamo:** conceptualization (equal), data curation (equal), formal analysis (equal), investigation (equal), writing – review and editing (equal). **Marta Chiaravalli:** conceptualization (equal), data curation (equal), formal analysis (equal), investigation (equal), writing – review and editing (equal). **Andrea Palloni:** conceptualization (equal), data curation (equal), formal analysis (equal), investigation (equal), writing – review and editing (equal). **Riccardo Carloni:** conceptualization (equal), data curation (equal), formal analysis (equal), investigation (equal), writing – review and editing (equal). **Alessandro Bittoni:** conceptualization (equal), data curation (equal), formal analysis (equal), investigation (equal), writing – review and editing (equal). **Monica Niger:** conceptualization (equal), data curation (equal), formal analysis (equal), investigation (equal), writing – review and editing (equal). **Ilario Giovanni Rapposelli:** conceptualization (equal), data curation (equal), formal analysis (equal), investigation (equal), writing – review and editing (equal). **Maria Grazia Rodriquenz:** conceptualization (equal), data curation (equal), formal analysis (equal), investigation (equal), writing – review and editing (equal). **Mario Scartozzi:** conceptualization (equal), data curation (equal), formal analysis (equal), investigation (equal), writing – review and editing (equal). **Stefania Mosconi:** conceptualization (equal), data curation (equal), formal analysis (equal), investigation (equal), writing – review and editing (equal). **Elisa Giommoni:** conceptualization (equal), data curation (equal), formal analysis (equal), investigation (equal), writing – review and editing (equal). **Ilaria Bernardini:** conceptualization (equal), data curation (equal), formal analysis (equal), investigation (equal), writing – review and editing (equal). **Chiara Paratore:** conceptualization (equal), data curation (equal), formal analysis (equal), writing – review and editing (equal). **Andrea Spallanzani:** conceptualization (equal), data curation (equal), formal analysis (equal), investigation (equal), writing – review and editing (equal). **Katia Bencardino:** conceptualization (equal), data curation (equal), formal analysis (equal), investigation (equal), writing – review and editing (equal). **Laura Forti:** conceptualization (equal), data curation (equal), formal analysis (equal), investigation (equal), writing – review and editing (equal). **Emiliano Tamburini:** conceptualization (equal), data curation (equal), formal analysis (equal), investigation (equal), writing – review and editing (equal). **Sara Lonardi:** conceptualization (equal), data curation (equal), formal analysis (equal), investigation (equal), writing – review and editing (equal). **Aldo Scarpa:** conceptualization (equal), formal analysis (equal), funding acquisition (equal), investigation (equal), methodology (equal), validation (equal), writing – review and editing (equal). **Stefano Cascinu:** conceptualization (equal), formal analysis (equal), investigation (equal), supervision (equal), writing – review and editing (equal). **Giampaolo Tortora:** conceptualization (equal), data curation (equal), formal analysis (equal), investigation (equal), writing – review and editing (equal). **Isabella Sperduti:** conceptualization (lead), data curation (lead), formal analysis (lead), methodology (lead), writing – original draft (equal), writing – review and editing (equal). **Michele Reni:** conceptualization (lead), data curation (equal), formal analysis (lead), investigation (lead), methodology (lead), writing – original draft (lead), writing – review and editing (lead).

## Ethics Statement

To undergo genetic testing at each participating institution, all patients signed an informed consent statement that permitted testing and data collection, analysis, and elaboration; due to this requirement, retrospective analysis of aggregated and irreversibly anonymized data was considered to be exempt from specific IRB approval.

## Conflicts of Interest

The authors declare the following financial interests/personal relationships which may be considered as potential competing interests: Michele Milella, Guido Giordano: personal honoraria from AstraZeneca, MSD; travel expenses from AstraZeneca. Giulia Orsi, Silvia Noventa, Silvia Bozzarelli, Ingrid Garajova, Enrico Vasile, Marina Macchini, Alessandro Cavaliere, Marina Gaule, Marta Chiaravalli, Andrea Palloni, Riccardo Carloni, Alessandro Bittoni, Maria Grazia Rodriquenz, Stefania Mosconi, Elisa Giommoni, Ilaria Bernardini, Chiara Paratore, Katia Bencardino, Aldo Scarpa, Stefano Cascinu, Isabella Sperduti: no competing interests. Mariacristina Di Marco: personal honoraria from OncoSil Medical; travel expenses from Viatris. Lisa Salvatore: personal honoraria from MSD, AstraZeneca, Servier, Bayer, Merck, Amgen, Pierre‐Fabre, Takeda, GSK. Letizia Procaccio: participation in advisory boards for AstraZeneca. Francesca Bergamo: participation in advisory boards for Servier, AAA, Novartis; personal honoraria from Eli‐Lilly, MSD, EISAI, Bayer, BMS, Amgen. Monica Niger: personal honoraria from Accademia di Medicina, Incyte, Sandoz, Medpoint SRL, Servier, EMD Serono, Basilea Pharmaceutica, MSD, Astrazeneca, Taiho; travel expenses from AstraZeneca. Ilario Giovanni Rapposelli: participation in Advisory Boards for MSD. Mario Scartozzi: personal honoraria from MSD, GSK, Servier, Astra Zeneca, Astellas, MERCK, Sanofi. Andrea Spallanzani: personal honoraria from Lilly, MSD, Astellas, Astrazeneca, Servier, Pierre Fabre. Laura Forti: participation in advisory boards for Amgen, Merck‐Serono, Servier. Emiliano Tamburini: participation in advisory boards for AstraZeneca, Amgen, Servier, and MSD. Sara Lonardi: research funding (to institution) from Amgen, Astellas, Astra Zeneca, Bayer, Bristol‐Myers Squibb, Daichii Sankyo, Hutchinson, Incyte, Merck Serono, Mirati, MSD, Pfizer, Roche, Servier; personal honoraria from Amgen, Bristol‐Myers Squibb, Incyte, GSK, Lilly, Merck Serono, MSD, Pierre‐Fabre, Roche, Servier; participation in advisory boards for Amgen, Astellas, Astra Zeneca, Bayer, Bristol‐Myers Squibb, Daiichi‐Sankyo, GSK, Incyte, Lilly, Merck Serono, MSD, Servier, Takeda. Giampaolo Tortora: participation in advisory boards for AstraZeneca, BMS, MSD, Merck, Servier. Michele Reni: participation in advisory boards for BMS, PANAVANCE, Viatris, SOTIO, Lilly, Servier, MSD, AstraZeneca, Celgene, Shire, Baxter; research funding (to institution) from AstraZeneca.

## Supporting information


Data S1.

## Data Availability

The data that support the findings of this study are available on request from the corresponding author. The data are not publicly available due to privacy or ethical restrictions.
