# Supplementary material for: Real‐World Impact of Olaparib Exposure in Advanced Pancreatic Cancer Patients Harboring Germline BRCA1‐2 Pathogenic Variants
Source: Cancer Med. 2025 Jan 24;14(3):e70364. doi: 10.1002/cam4.70364 (PMC11761426; doi:10.1002/cam4.70364)
Supplement: Supplementary file 1 — Data S1. [file CAM4-14-e70364-s001.docx]

***Supplementary materials***

**Supplementary Table S1: OS in g*BRCA*1-2pv PDAC patients exposed or not to olaparib (any treatment line).**

| **Cohort** | **Olaparib** | **mOS**  **(mos; 95% CI)** | **Log-rank**  ***p-*value** | **HR (95% CI)** |
| --- | --- | --- | --- | --- |
| **Entire cohort (n=114)** | No (n=61) | 16 (12-20) | 0.02 | 0.568 (0.351-0.918) |
|  | *Yes (n=53)* | *34 (24-44)* |  |  |
| **M+ (n=87)** | No (n=47) | 14 (11-18) | 0.0025 | 0.445 (0.260-0.762) |
|  | *Yes (n=40)* | *34 (25-43)* |  |  |
| **No PD (n=94)** | No (n=43) | 17   (8-25) | 0.049 | 0.585 (0.341-1.014) |
|  | *Yes (n=50)* | *34 (24-43)* |  |  |
| **M+/no PD (n=73)** | No (n=35) | 16 (12-20) | 0.057 | 0.598 (0.315-1.026) |
|  | *Yes (n=38)* | *34 (24-44)* |  |  |

mOS: median overall survival (in months - mos); 95% CI: 95% confidence interval); HR: hazard ratio; M+: g*BRCA* PDAC patients with stage IV disease at the time of diagnosis; no PD: g*BRCA* PDAC patients who did not experience PD as their best response to I-line chemotherapy; M+/no PD: M+ g*BRCA* PDAC patients who did not experience PD as their best response to I-line chemotherapy.

**Supplementary Table S2: Univariate and multivariate Cox regression analysis for OS (all patients).**

| **Factor** | ***Univariate*** | | ***Multivariate*** | |
| --- | --- | --- | --- | --- |
|  | **HR (95% CI)** | ***p*-value** | **HR (95% CI)** | ***p*-value** |
| **Age** | 1.019 (0.994-1.044) | 0.141 | - |  |
| **Gender (female vs male)** | 0.996 (0.612-1.621) | 0.996 | - |  |
| **Stage at Diagnosis (I-III vs IV)** | 0.653 (0.346-1.232) | 0.653 | - |  |
| ***CA19.9 (below median vs above)*** | *0.421 (0.246-0.721)* | *0.002* | *0.546 (0.313-0.953)* | *0.033* |
| **Neodjuvant CHT (yes vs no)** | 0.885 (0.403-1.944) | 0.760. | - |  |
| ***Surgery (yes vs no)*** | *0.296 (0.131-0.553)* | *<0.0001* | *0.335 (0.150-0.748)* | *0.008* |
| **Adjuvant CHT (yes vs no)** | 0.434 (0.182-1.032) | 0.059 | - |  |
| **Platinum (yes vs no)** | 0.769 (0.474-1.249) | 0.289 | - |  |
| ***Response to I-line***  ***CR/PR vs PD***  ***SD vs PD***  ***CR/PR vs SD*** | *-*  *0.433 (0.232-0.806)*  *0.754 (0.371-1.531)*  *0.574 (0.324-1.018)* | *0.016*  *0.008*  *0.434*  *0.057* | *-*  *0.347 (0.182-0.664)*  *0.552 (0.263-1.157)*  *0.629 (0.342-1.158)* | *0.005*  *0.001*  *0.116*  *0.137* |
| **Any olaparib (yes vs no)** | 0.568 (0.351-0.918) | 0.021 | - |  |
| **Maintenance olaparib (yes vs no)*** | 0.557 (0.336-0.923) | 0.023 | - |  |

HR: hazard ratio; 95% CI: 95% confidence interval; CHT: chemotherapy; PD: progressive disease; CR: complete response; PR: partial response; SD: stable disease.

*Patients receiving olaparib as maintenance therapy in the absence of progression to chemotherapy in any treatment line (n=43) were considered.

**Supplementary Table S3: Baseline characteristics of g*BRCA*1-2pv PDAC patients receiving olaparib as second or third/further line of therapy, upon progression to the previous line of chemotherapy.**

| **Patients' characteristics**  **(N=10)** | **N** |
| --- | --- |
|  |  |
| **Age (yrs)** | Median: 56  Range: 46-76 |
| **Gender**  **M**  **F** | 4  6 |
| **Metastatic disease at diagnosis**  **Yes**  **No** | 8  2 |
| **Liver mets**  **Yes**  **No** | 5  5 |
| **Previous surgery**  **Yes**  **No** | 3  7 |
| **Platinum-based CHT**  **Any**  **I line**  **II or subsequent**  **Immediately before olaparib** | 9  4  5  4 |
| **Best response to I-line**  **CR/PR**  **SD**  **PD** | 6  2  2 |

**
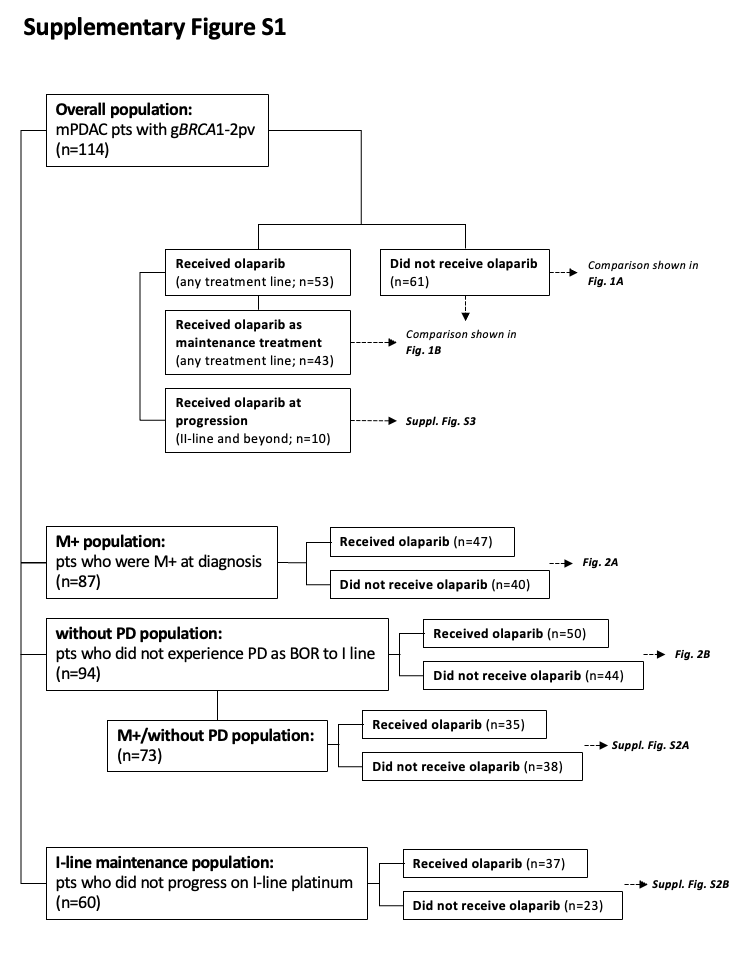
**

**
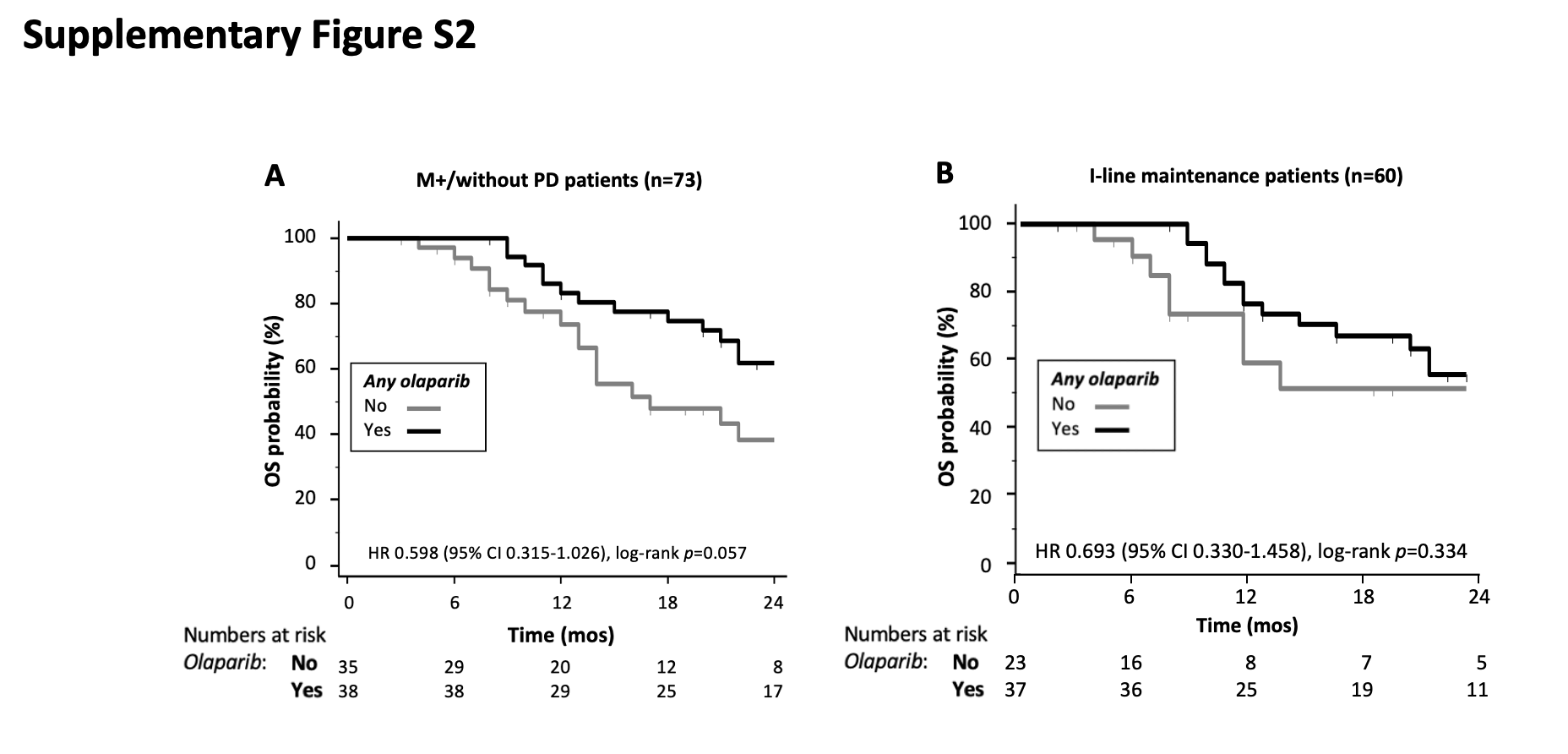
**

**
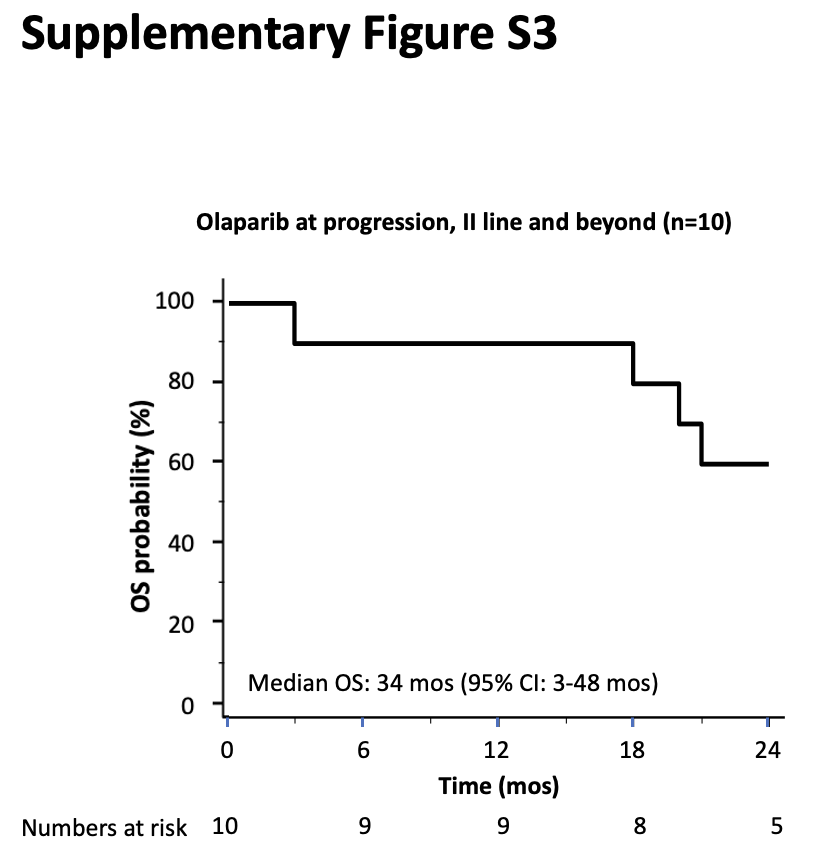
**

**Supplementary Figure legends.**

**Supplementary Figure S1: Flowchart of patients' selection criteria for each individual comparison.** Among 114 patients with documented g*BRCA*1-2pv (**Overall population**), 61 patients never received olaparib and a total of 53 patients received olaparib regardless of the treatment line (see also **Tables 1** and **2**). Among the latter, 43 received olaparib as maintenance treatment (i.e., in the absence of PD to the immediately preceding chemotherapy line: I-, II-, and III-line maintenance in 37, 5, and 1 patient, respectively; see also **Table 2**); 10 patients received olaparib after progression to the immediately preceding chemotherapy line (II- and III-line of treatment for metastatic disease in 2 and 8 patients, respectively; see also **Table 2**). Given the strong prognostic impact of previous surgery and response to I-line chemotherapy, we also analyzed patients with stage IV disease at first diagnosis (**M+ population**) and patients who did not experience PD as their best overall response (BOR; **without PD population**) separately; patients with both stage IV disease at first diagnosis and no PD as their best response to I-line chemotherapy formed the **M+/without PD population** (see also **Supplementary Table S1**). Finally, patients matching the inclusion criteria of the POLO trial (*patients who had a germline BRCA1 or BRCA2 mutation, metastatic pancreatic cancer, and disease that had not progressed during first-line platinum-based chemotherapy,* ref. 11) were analyzed as **I-line maintenance population***.*

**Supplementary Figure S2: Impact of olaparib exposure in subgroups of patients closer to POLO trial patient population. A.** Kaplan-Meier curves for OS in g*BRCA*1-2pv PDAC patients with stage IV disease at the time of diagnosis (M+) and who did not experience PD as their best response to first-line chemotherapy (no PD), according to having (*yes*, black line; n=38) or not having (*no*, grey line; n=35) received olaparib, are shown; HR with 95% CI and statistical significance of the differences between curves according to log-rank test are reported. **B**. Kaplan-Meier curves for g*BRCA*1-2pv PDAC patients not progressing on platinum-based first-line chemotherapy, who received (*yes*, black line; n=37) or did not receive (*no*, grey line; n=23) maintenance olaparib (POLO trial population), are shown; HR with 95% CI and statistical significance of the differences between curves according to log-rank test are reported.

**Supplementary Figure S3: Survival of advanced g*BRCA*1-2pv PDAC patients receiving olaparib after progression to a previous line of chemotherapy.** Kaplan-Meier curve for OS for the 10 patients who received olaparib as second (n=2) or third and further (n=8) line of systemic treatment for metastatic disease, immediately after progressing to the previous chemotherapy line, is shown; median OS with 95% CI is reported.
